# Minimally invasive surgery alone compared with intensity-modulated radiotherapy for primary stage I nasopharyngeal carcinoma

**DOI:** 10.1186/s40880-019-0415-3

**Published:** 2019-11-15

**Authors:** You-Ping Liu, Xing Lv, Xiong Zou, Yi-Jun Hua, Rui You, Qi Yang, Le Xia, Shao-Yan Guo, Wen Hu, Meng-Xia Zhang, Si-Yuan Chen, Mei Lin, Yu-Long Xie, Li-Zhi Liu, Rui Sun, Pei-Yu Huang, Wei Fan, Xiang Guo, Ming-Huang Hong, Ming-Yuan Chen

**Affiliations:** 1 Department of Nasopharyngeal Carcinoma, Sun Yat-sen University Cancer Center, 651 Dongfeng East Road, Guangzhou, 510060 Guangdong P. R. China; 2 State Key Laboratory of Oncology in South China, Collaborative Innovation Center for Cancer Medicine, Guangdong Key Laboratory of Nasopharyngeal Carcinoma Diagnosis and Therapy, Sun Yat-sen University Cancer Center, Guangzhou, 510060 Guangdong P. R. China; 3 Department of Medical Imaging and Interventional Radiology, Sun Yat-sen University Cancer Center, Guangzhou, 510060 Guangdong P. R. China; 4 Department of Nuclear Medicine, Sun Yat-sen University Cancer Center, Guangzhou, 510060 Guangdong P. R. China; 5 Department of Clinical Trials Center, Sun Yat-sen University Cancer Center, Guangzhou, 510060 Guangdong P. R. China

**Keywords:** Nasopharyngeal carcinoma, Localized, Early stage, Endoscopic nasopharyngectomy, Intensity-modulated radiotherapy, Survival, Medical cost, Quality of life

## Abstract

**Background:**

The National Comprehensive Cancer Network guidelines recommend intensity-modulated radiotherapy (IMRT) as the primary curative treatment for newly diagnosed nasopharyngeal carcinoma (NPC), but the radiation-related complications and relatively high medical costs remain a consequential burden for the patients. Endoscopic nasopharyngectomy (ENPG) was successfully applied in recurrent NPC with radiation free and relatively low medical costs. In this study, we examined whether ENPG could be an effective treatment for localized stage I NPC.

**Methods:**

Ten newly diagnosed localized stage I NPC patients voluntarily received ENPG alone from June 2007 to September 2017 in Sun Yat-sen University Cancer Center. Simultaneously, the data of 329 stage I NPC patients treated with IMRT were collected and used as a reference cohort. The survival outcomes, quality of life (QOL), and medical costs between two groups were compared.

**Results:**

After a median follow-up of 59.0 months (95% CI 53.4–64.6), no death, locoregional recurrence, or distant metastasis was observed in the 10 patients treated with ENPG. The 5-year overall survival, local relapse-free survival, regional relapse-free survival, and distant metastasis-free survival among the ENPG-treated patients was similar to that among the IMRT-treated patients (100% vs. 99.1%, 100% vs. 97.7%, 100% vs. 99.0%, 100% vs. 97.4%, respectively, *P *> 0.05). In addition, compared with IMRT, ENPG was associated with decreased total medical costs ($ 4090.42 ± 1502.65 vs. $ 12620.88 ± 4242.65, *P *< 0.001) and improved QOL scores including dry mouth (3.3 ± 10.5 vs. 34.4 ± 25.8, *P *< 0.001) and sticky saliva (3.3 ± 10.5 vs. 32.6 ± 23.3, *P *< 0.001).

**Conclusions:**

ENPG alone was associated with promising long-term survival outcomes, low medical costs, and satisfactory QOL and might therefore be an alternative strategy for treating newly diagnosed localized stage I NPC patients who refused radiotherapy. However, the application of ENPG should be prudent, and prospective clinical trials were needed to further verify the results.

## Background

Nasopharyngeal carcinoma (NPC) is rare worldwide but common in China, Southeast Asia, and North Africa, with the highest incidence in Southern China [1–3]. Unlike other head and neck cancers, NPC is considered “unresectable” due to high-frequency of extra-cavity involvement, regional metastasis at diagnosis and the difficulty of the surgical approach to the nasopharynx. Radiotherapy is currently regarded as the only curative option for stage I NPC according to National Comprehensive Cancer Network (NCCN) guidelines. Although mortality rates among patients with stage I NPC treated with intensity-modulated radiotherapy (IMRT) are less than 5% [4, 5], almost all patients develop mild to moderate acute toxicities, including mucositis, pharyngitis and xerostomia, with subsequent consequences for quality of life (QOL) during convalescence [5, 6]. In addition, the low cost effectiveness and late complications of IMRT cannot be ignored.

With the popularity of health education and the development of early cancer screening in NPC, an increasing number of NPC patients with early stage cancer were screened and diagnosed. 17.3% to 47.1% of patients were found in stage I [7, 8], with the lesion limited in the nasopharyngeal cavity in the early screening population, allowing them to be radically and surgically resected. Furthermore, with the development of nasal endoscopic techniques in recent decades, the endoscopic endonasal approach (EEA) allows surgeons to treat many deeply located tumors, even those once considered “inoperable”, without functional disability. We developed a novel endoscopic nasopharyngectomy (ENPG) [9–11] and applied this technique in recurrent NPC (rNPC) in 2004 [12–14]. Our previous studies have shown that salvage ENPG resulted in better overall survival (OS) and less posttreatment complications and medical costs than salvage IMRT [13].

These results suggest that it is theoretically possible to radically excise newly diagnosed localized stage I NPC lesions with ENPG and avoid radiation-related toxicities. Here, we report our initial experience with ENPG alone in localized stage I NPC, aiming to assess the efficiency, microinvasion and cost effectiveness of ENPG as an alternative for IMRT in localized stage I NPC.

## Methods

### Patient selection

A total of 490 patients who were newly diagnosed with stage I NPC were identified from an inpatient database at the Sun Yat-sen University Cancer Center (Guangzhou, China) from June 2007 to September 2017.

Our NPC research team, consisting of head and neck surgeons, radiation oncologists, medical oncologists, pathologists, radiologists, and nuclear medicine physicians, established the indication, relative contraindication, and absolute contraindication criteria for applying ENPG in the localized stage I NPC.

The indication criteria for ENPG were: (1) had a maximum diameter of the primary tumor ≤ 1.5 cm; (2) distance of the tumor margin to the internal carotid artery was ≥ 0.5 cm; (3) the minimal axial diameter in magnetic resonance imaging (MRI) was no more than 0.4 cm for retropharyngeal lymph nodes (RPLN) and 0.6 cm for cervical lymph node (CLN). Relative contraindications criteria for ENPG were: (1) exogenous tumor diameter > 1.5 cm but tumor basis ≤ 1.5 cm; (2) RPLN and CLN ranged 0.4–0.5 cm and 0.6–1.0 cm, respectively, for minimal diameter but was proved negative by ^18^F-fluorodeoxyglucose positron emission tomography and computed tomography (PET/CT) or pathology. Absolute contraindications criteria for ENPG were: (1) tumor basis diameter > 1.5 cm, or occupied the entire nasopharyngeal cavity; (2) T2–T4 primary tumor, i.e. tumor involving to or beyond the pharyngobasilar fascia; (3) N1–N3 regional lymph node metastasis. i.e. LN with central necrosis or annular enhancement, or groups of two or more lymph nodes (the minimal diameter ≥ 8 mm for CLN), or minimal diameter ≥ 0.5 cm for RPLN, or minimal diameter ≥ 1.0 cm for CLN; (4) had distant metastasis, i.e. bone, liver, or lung metastasis; (5) was physiologically unsuitable for surgery.

In the present study, the inclusion and exclusion criteria for applying ENPG were as follows:

Inclusion criteria: (1) all patients were previously untreated, pathologically diagnosed with undifferentiated or differentiated, keratinizing or nonkeratinizing NPC; (2) staged as T1N0M0 classification; stage I according to the 8th edition staging system of the American Joint Committee on Cancer [AJCC], referring to primary tumors confined in the nasopharyngeal cavity, and the RPLN and CLN were no more than 0.5 cm and 1.0 cm respectively. Of note, the inclusion criteria also comprised of patients with relative contraindications. Exclusion criteria: (1) in accordance with the absolute contraindications criteria; (2) without intention to surgery.

All patients provided preoperative written informed consent. The study was approved by the ethical committee of Sun Yat-Sen University Cancer Center.

### Treatment procedures

The detailed procedures for administrating ENPG were performed as previously reported [9, 10]. All operations were performed under systemic anesthesia with an electrotome guided by a 4-mm rigid endoscope (0° and 30°, Karl-Storz, Tuttlingen, Germany).

Before the operation, our NPC research team first defined the tumor invasion regions and surgical margins for high-risk microinvasion regions, such as gross tumor volume (GTV) and high risk clinical target volume (CTV1) in radiotherapy, respectively [15]. In principle, surgical margins were defined as the tumor invasion regions plus an additional 0.5–1.0 cm peripheral mucosal margin and a 2–3 mm basal margin on the surface of the sphenoid bone and the clivus in the skull base (Fig. 2a–c). The surgeons were required to strictly follow this planned surgical boundary to remove the tumor during the dissection.

Commonly, the posterior column of the nasal septum was first removed, then the mucoperiosteum in the roof wall of the nasopharynx was separated from the surface of the sphenoid to the clivus. After excising the bilateral eustachian cartilage from the pharyngeal recess under the mucous membrane and foramen lacerum, we separated the posterior nasopharyngeal mucoperiosteum along the clivus. After that, the mucoperiosteum at the level of the soft palate from the surface of the prevertebral muscle was isolated and subsequently turned upward to meet the other resection margins. Thus, the whole nasopharyngeal cavity mucosa including the swelling around the tumor was removed en bloc. To recover the defect, the nasal septum and floor mucosa were separated from the surface of the bone, and only a narrow posterior pedicle above the posterior naris remained to contain the posterior septal artery. The flap was then gently rotated backward and unrolled to cover the nasopharyngeal defect.

Radiotherapy was administered with IMRT techniques. Target volumes definition were as previously reported [15]. The prescribed dose was 66–70 Gy, 60–62 Gy, and 54–56 Gy, in 28–33 fractions, for the planning target volumes (PTVs) derived from GTV, CTV1, and the low-risk clinical target volume (CTV2), respectively. GTV was determined by physical examination, imaging (including MRI and PET/CT, if available) and endoscopic findings. CTV1 was defined as the GTV region plus an additional anterior, superior, inferior and lateral margin of 5 mm to 1 cm and an additional posterior margin of 2 mm to 3 mm (the range of extension was determined by adjacent structural characteristics), the CTV1 volume also included the entire mucosal stratum and 5 mm of submucosal stratum of the nasopharynx. The CTV2 was defined as the CTV1 region plus an additional anterior, superior, inferior, and lateral margin of 5 mm to 10 mm and an additional posterior margin of 2 mm to 3 mm, and bilateral upper neck lymph node groups that were at risk of potential microscopic spread of disease [15]. Simultaneously integrated boost, with 5 fractions per week, was adopted.

### Operation assessments

The success of the operation was evaluated by the NPC research team, based on the following three conditions: (1) the intraoperative macroscopic observation showed no tumor-like tissues residue (Fig. 2d, h); (2) had negative margins on pathology; and (3) underwent total resection of the planned resection volume, judged by comparison of preoperative magnetic resonance imaging (MRI) to postoperative, within 1 week after the surgery (Fig. 2a–c, e, f, g).

### Quality of life and cost effectiveness

All patients were asked to complete the European Organization for Research and Treatment of Cancer Quality of Life Questionnaire-Core 30 general (EORTC QLQ-C30, version 3) and head and neck-specific (EORTC QLQ-H&N35) questionnaires in the latest follow-up [16]. Medical cost data were provided by the financial department of our cancer center, which included examination expenses, hospital bed cost, nursing care, anesthesia, medicine, radiotherapy, operation, blood transfusion, and other treatments related costs ($1.00 = ¥6.72 [11th March 2019]).

### Follow-up

The last follow-up date was on May 17, 2019. During the follow-up, the patients usually underwent endoscopies to assess the wound reconstruction every 2 weeks until the wound was completely re-epithelialized. Subsequent follow-up assessments were performed every 3 months during the first year and every 6 months thereafter until the fifth year. Nasopharyngoscopy, MRI of the head and neck, chest radiography, and abdominal sonography were performed at each assessment. Whenever possible, salvage treatments, including radiotherapy, chemotherapy, and surgery were administered to patients after tumor relapse.

### Statistical analysis

Categorical variables were analyzed using the χ^2^ test, Fisher’s exact test and continuous variables were analyzed using the Mann–Whitney U test. The events for OS, distant metastasis-free survival (DMFS), local relapse-free survival (LRFS) and regional relapse-free survival (RRFS) were death from any cause, distant metastasis, local and regional relapse, respectively. The duration was calculated from the date of diagnosis for NPC to the date of each event or the last follow-up. Survival results were calculated using the Kaplan–Meier method, and differences were compared by log-rank test. All analyses were performed using Statistical Product and Service Solutions (SPSS) software (version 22.0, SPSS Inc., Chicago, USA), and a 2-tailed *P* < 0.05 was considered as statistically significant.

## Results

### Patients

**Fig. 1:**
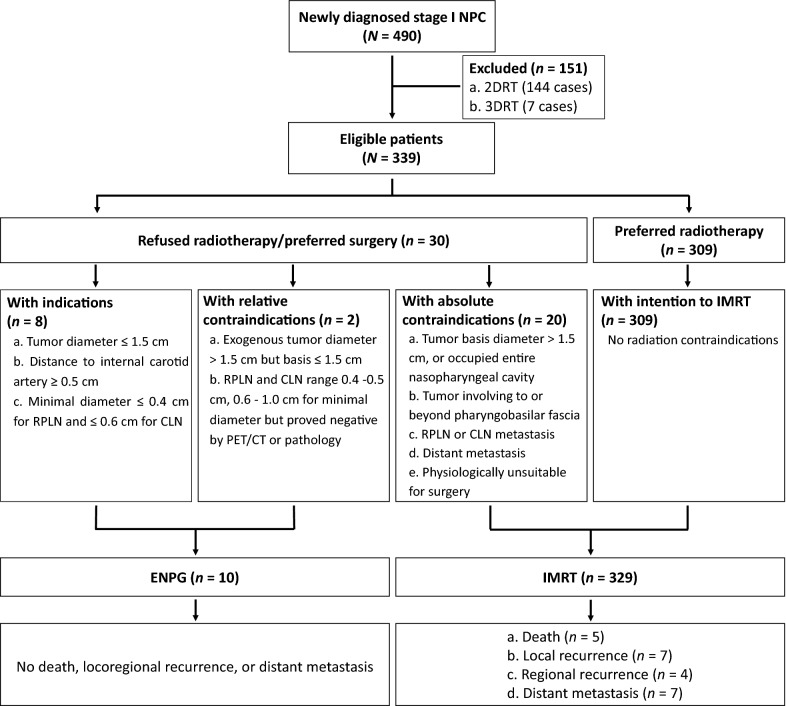
Work flow diagram. *NPC* nasopharyngeal carcinoma, *2DRT* two-dimensional radiotherapy, *3DRT* three-dimensional radiotherapy, *RPLN* retropharyngeal lymph node, *CLN* cervical lymph node, *PET/CT* ^18^F-fluorodeoxyglucose positron emission tomography and computed tomography, *ENPG* endoscopic nasopharyngectomy, *IMRT* intensity-modulated radiotherapy

From June 2007 to September 2017, a total of 339 patients were found to be eligible after exclusion of patients who underwent two-dimensional radiotherapy (2DRT) and three-dimensional radiotherapy (3DRT). Patients who refused radiotherapy or preferred surgery were treated with ENPG alone if they fit the inclusion criteria, and the rest were treated with IMRT (Fig. 1). Finally, 10 patients with localized stage I NPC accepted the ENPG treatment voluntarily and 329 patients (309 with intension to radiotherapy and 20 with absolute contraindications for ENPG) underwent IMRT. The mean age of the 10 patients with ENPG and 329 patients with IMRT were 47.0 years (range 29 to 73 years) and 45.0 years (range 19 to 75 years), respectively. Table 1 outlined the demographic and clinical characteristics (Table 1). Due to a controversial lymph node in the level II of left neck, which had a maximum minimal diameter of 0.8 cm in MRI but slightly higher ^18^F-FDG uptake in PET/CT, one patient underwent lymphadenectomy before ENPG, and the pathology was confirmed as negative of tumor involvement.

**Fig. 2:**
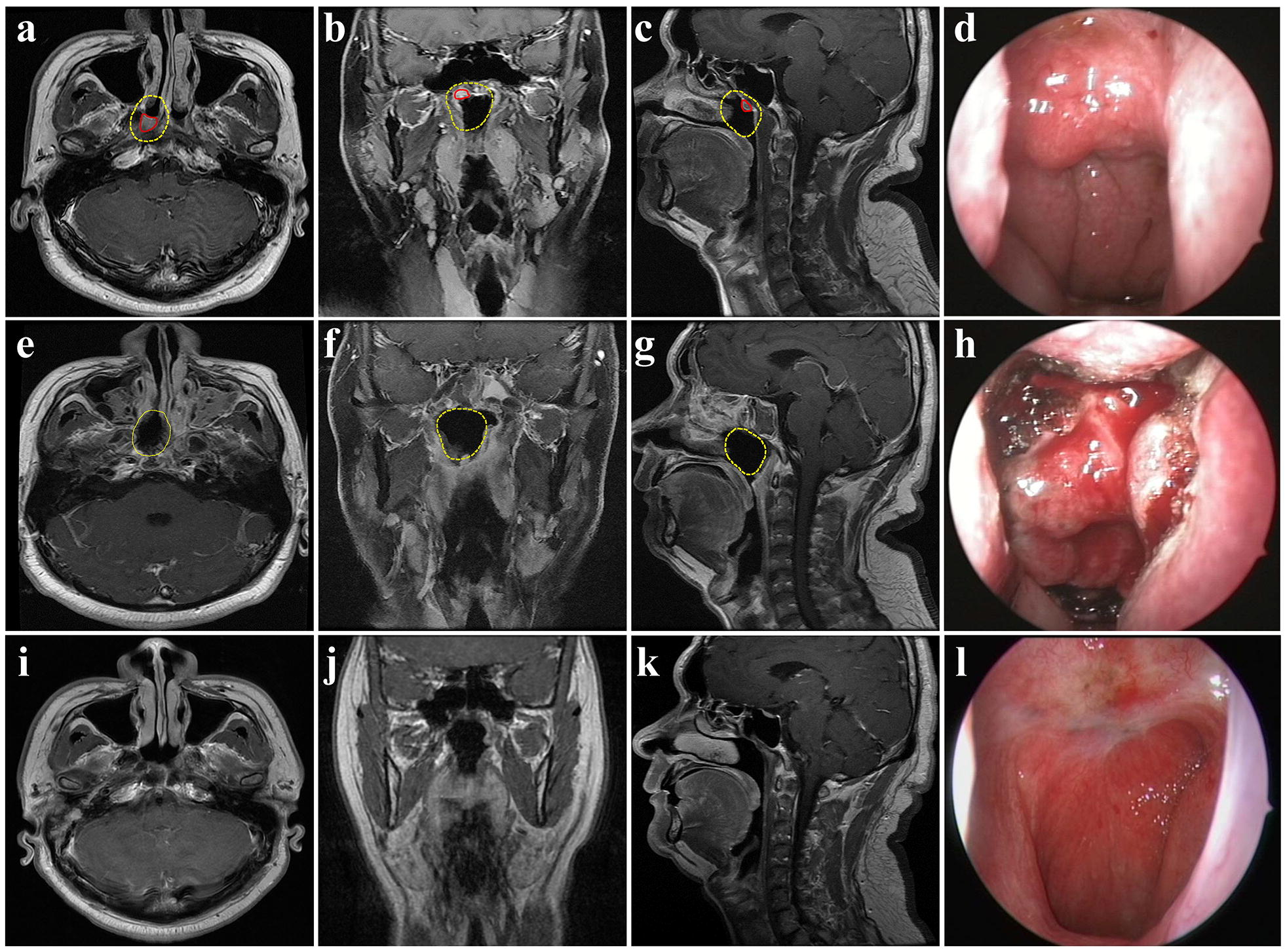
Magenetic resonance and endoscopic images contrast before and after surgery. **a**–**c** shown the tumor invasion regions (red line) and surgical margin (yellow line) in preoperative T1-weighted MR images in horizontal, coronal and sagittal view, respectively. The tumor invasion regions were located in the right superior wall of the nasopharynx and the planed surgical margin which was made by our NPC experts team before surgery covered most of the superior wall and part of the posterior wall of the nasopharynx. **d** was the preoperative endoscopic nasopharyngeal image and shown that nodular masses were found in the right superior wall of the nasopharynx, not involving the bilateral pharyngeal recesses and eustachian tubes. **e**–**g** shown the resection regions in T1-weighted MR images in horizontal, coronal and sagittal view, respectively, at 3 days after surgery. The surgical defect was highly similar to the planned surgical margin (yellow line). **h** shown the endoscopic nasopharynx images during operation, which was marking the resection boundary according to the planed surgical margin delinerated before surgery. **i**–**k** shown no abnormal neoplasm was observed in nasopharynx in T1-weighted MR images in horizontal, coronal and sagittal view, respectively, at 10 years after surgery. **l** shown the synchronous endoscopic nasopharyngeal image and shown the nasopharyngeal defect was re-epithelized and no abnormal neoplasm was observed

**Table 1:** Characteristics of stage I patients with ENPG and IMRT

Variable	ENPG	IMRT	*P*
	*n* (%)	*n* (%)	
Gender			0.466*
Female	3 (30.0)	72 (21.9)	
Male	7 (70.0)	257 (78.1)	
Age [median + SD (range)]	47.0 ± 14.5 (29–73)	45.0 ± 10.9 (19–75)	0.480^#^
BMI [mean + SD (range)]	24.4 ± 2.7 (18.8–27.9)	23.2 ± 3.0 (15.6–33.4)	0.129^#^
Karnofsky performance status			0.215*
≥ 90	9 (90.0)	322 (97.9)	
< 90	1 (10.0)	7 (2.1)	
Clinical stage			–
I	10 (100.0)	329 (100.0)	
II	0	0	
III	0	0	
IV	0	0	
Histology			0.306*
WHO I	0	0	
WHO II	1 (10.0)	11 (3.3)	
WHO III	9 (90.0)	318 (96.7)	
Smoking history			0.461*
Yes	1 (10.0)	84 (25.5)	
No	9 (90.0)	245 (74.5)	
Total doses [mean + SD (range)]	–	68.85 ± 1.24 (66.00–70.29)	–
Fractionated dose [mean + SD (range)]	–	2.24 ± 0.08 (2.00–2.36)	–
Reconstruction		–	–
Yes	6 (60.0)	–	
No	4 (40.0)	–	

*ENPG* endoscopic nasopharyngectomy, *IMRT* intensity-modulated radiotherapy, *SD* standard deviation

^*^ Fisher’s exact test

^#^Mann–Whitney U test

### Surgical treatment characteristics

All operations were safely performed as minimally invasive procedures without any severe surgery-related complications. The median duration of surgery was 92.5 min (range 60 to 135 min). The median quantity of bleeding was 20 mL (range 10 to 100 mL). No patients required blood transfusion. All the multiple margins of biopsies were pathologically negative, and postoperative MRI revealed that all the planned surgical margins of the patients with ENPG were completely resected after comparison to preoperative MRI. Thus, surveillance but not adjuvant radiotherapy or chemotherapy was advised for the 10 ENPG-treated patients. Four patients without nasal flap reconstruction in the operation complained of neck-stiffness-like discomfort or a slight headache. These symptoms disappeared after the surgical defect re-epithelized nearly 1 month after surgery. To alleviate such impacts, the pedicle nasal flap technique was applied for the remaining six patients. The patients did not complain of such discomfort after surgery, and their defects re-epithelized within 2 weeks.

### Survival outcomes

The last follow-up date was May 17, 2019. After a median follow-up of 59.0 months (95% CI 53.4–64.6), no patients developed tumor recurrence or metastasis, and no death was observed among the 10 patients with ENPG. One of them had a squamous cell carcinoma of the trachea at 5 years after the ENPG surgery. He underwent concurrent chemoradiotherapy for trachea carcinoma. The tumor achieved a complete response and had no progression at last follow-up.

**Fig. 3:**
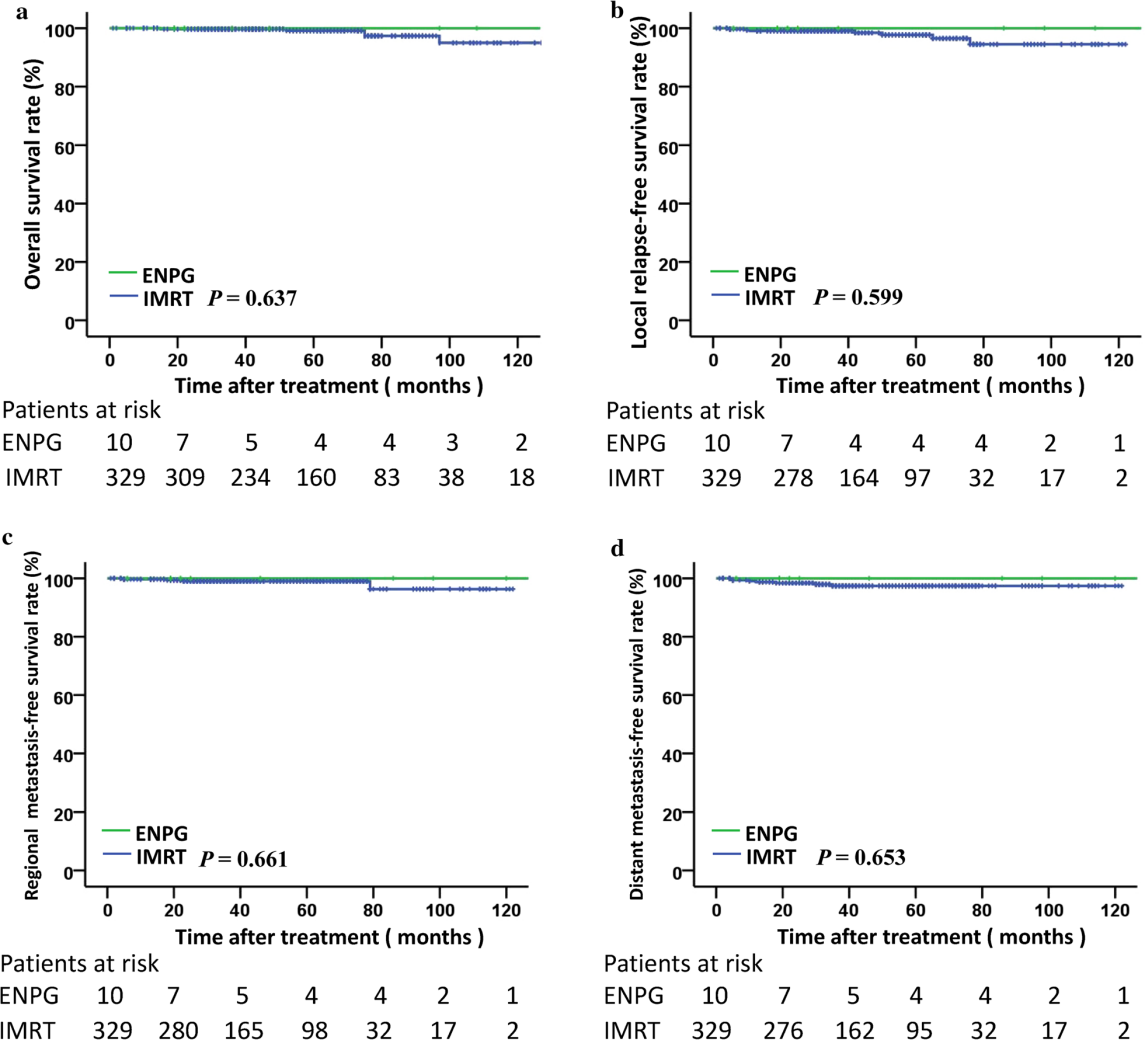
Kaplan–Meier curves of overall survival (**a**), local relapse-free survival (**b**), regional relapse-free survival (**c**) and distant metastasis-free survival (**d**) for stage I NPC patients with ENPG and IMRT. *ENPG* endoscopic nasopharyngectomy, *IMRT* intensity modulated radiation therapy

The 5-year OS, LRFS, RRFS and DMFS among the patients with ENPG was 100% and those with IMRT in the reference cohort were 99.1%, 97.7%, 99.0% and 97.4%, respectively, which were similar to those among patients with ENPG (*P *> 0.05) (Fig. 3).

### Life quality outcomes and cost effectiveness

A total of 10 patients who underwent ENPG and 220 patients who underwent IMRT completed the questionnaires. ENPG was found to be slightly better than IMRT in the score assessment regarding items in QLQ-C30 and QLQ-H&N35, especially in terms of pain (0 ± 0 vs. 4.1 ± 7.4, *P* = 0.042), swallowing (0 ± 0 vs. 11.4 ± 18.5, *P* = 0.016), dry mouth (3.3 ± 10.5 vs. 34.4 ± 25.8, *P* < 0.001) and sticky saliva (3.3 ± 10.5 vs. 32.6 ± 23.3, *P* < 0.001) (Table 2). All 10 patients who underwent ENPG were alive without obvious late complications (Table 3).

**Table 2:** Inquiry of quality of life using the QLQ-C30, QLQ-H&N35

EORTC scale score	ENPG (*N* = 10)	IMRT (*N* = 220)	*P*
	Mean ± SD	Mean ± SD	
QLQ-C30			
Global health status	99.2 ± 2.6	96.5 ± 11.5	0.638
Functional scales			
Physical functioning	98.7 ± 2.8	96.6 ± 14.4	0.738
Role functioning	100	97.4 ± 14.9	0.568
Emotional functioning	99.2 ± 2.6	98.5 ± 7.0	0.643
Cognitive functioning	96.7 ± 7.0	97.7 ± 8.3	0.336
Social functioning	100	97.4 ± 14.5	0.540
Symptom scales			
Fatigue	1.1 ± 3.5	2.2 ± 12.0	0.402
Nausea and vomiting	0	0.2 ± 2.5	0.763
Pain	0	2.3 ± 10.2	0.449
Dyspnea	0	0.6 ± 4.5	0.668
Insomnia	0	1.7 ± 9.1	0.540
Appetite loss	0	8.3 ± 15.8	0.086
Constipation	0	0.3 ± 3.2	0.763
Diarrhea	0	0.2 ± 2.2	0.831
Financial difficulties	0	2.4 ± 14.4	0.568
QLQ-H & N35			
Pain	0	4.1 ± 7.4	*0.042*
Swallowing	0	11.4 ± 18.5	*0.016*
Senses problems	3.3 ± 10.5	2.3 ± 7.8	0.884
Speech problems	0	1.7 ± 5.6	0.307
Social eating	1.7 ± 3.5	4.3 ± 9.6	0.431
Social contact	0	1.2 ± 7.0	0.568
Sexuality	0	2.9 ± 12.4	0.394
Teeth	3.3 ± 10.5	5.5 ± 15.0	0.749
Opening mouth	0	5.5 ± 12.4	0.165
Dry mouth	3.3 ± 10.5	34.4 ± 25.8	< *0.001*
Sticky saliva	3.3 ± 10.5	32.6 ± 23.3	< *0.001*
Coughing	0	0.8 ± 5.0	0.631
Felt ill	0	3.3 ± 17.1	0.515
Pain killers	0	2.7 ± 18.9	0.631
Nutritional supplements	0	3.6 ± 23.1	0.598
Feeding tube	0	0.5 ± 6.7	0.831
Weight loss	10.0 ± 31.6	10.0 ± 45.7	0.572
Weight gain	0	2.7 ± 16.3	0.597

Italic values indicate significance of *P* value (*P* < 0.05)

*EORTC* the European Organization for Research and Treatment of Cancer, *QLQ*-*C30* Quality-of-Life Questionnaire–Core 30 general, *QLQ*-*H&N35* head and Neck–specific questionnaires, *ENPG* endoscopic nasopharyngectomy, *IMRT* intensity-modulated radiotherapy, *SD* standard deviation

**Table 3:** Late treatment-related complications

Complications	ENPG (*N* = 10)		IMRT (*N* = 220)		P*
	*n*	%	*n*	%	
Auditory/hearing					0.069
0	10	100	138	62.7	
1–2	0	0	63	28.7	
3–4	0	0	19	8.6	
Trismus					0.369
0	10	100	184	83.6	
1–2	0	0	36	16.4	
3–4	0	0	0	0	
Dysphagia					0.674
0	10	100	190	86.4	
1–2	0	0	26	11.8	
3–4	0	0	4	1.8	
Skin					1.000
0	9	90.0	193	87.7	
1–2	1	10.0	25	11.4	
3–4	0	0	2	0.9	
Subcutaneous soft tissue					0.611
0	10	100	193	87.7	
1–2	0	0	27	12.3	
3–4	0	0	0	0	
Dry mouth					< 0.001
0	9	90.0	52	23.6	
1–2	1	10.0	119	54.1	
3–4	0	0	49	22.3	
Cranial neuropathy					0.473
0	10	100	207	94.1	
1–2	0	0	13	5.9	
3–4	0	0	0	0	
Peripheral neuropathy					1.000
0	10	100	218	99.1	
1–2	0	0	2	0.9	
3–4	0	0	0	0	
Endocrine dysfunction					1.000
0	10	100	211	95.9	
1–2	0	0	9	4.1	
3–4	0	0	0	0	
Temporal lobe necrosis					1.000
0	10	100	205	93.2	
1–2	0	0	15	6.8	
3–4	0	0	0	0	

*ENPG* endoscopic nasopharyngectomy, *IMRT* intensity-modulated radiotherapy

* Fisher’s exact test

The direct cost and total treatment cost for these 10 patients with ENPG were $1260.60 ± 636.48 and $4090.42 ± 1502.65, which were obviously less than those for patients who underwent IMRT ($9647.39 ± 2676.09 and $12,620.88 ± 4242.65, *P* < 0.001) (Table 4).

**Table 4:** Medical cost in hospital stay

Variables	ENPG (*n* = 10)		IMRT (*n* = 329)		*P*
	Mean ± SD	Median	Mean ± SD	Median	
Indirect cost ($)	2829.82 ± 1268.38	3146.43	2973.49 ± 3267.11	2079.57	0.890
Hospital bed and nurse cost	193.79 ± 133.44	153.29	226.86 ± 251.18	94.12	0.472
Total workup	1126.13 ± 785.92	934.75	1160.24 ± 742.21	1039.26	0.886
Medicine	1293.68 ± 641.15	1183.52	1350.54 ± 2734.17	507.27	0.948
Others	216.23 ± 154.83	219.24	235.85 ± 1186.06	83.53	0.958
Direct cost ($)	1260.60 ± 636.48	1176.97	9647.39 ± 2676.09	9482.77	< 0.001
Costs of surgery/anesthesia	1260.60 ± 636.48	1176.97	12.68 ± 25.21	2.23	< 0.001
Radiation session IMRT	0	0	9634.71 ± 2673.39	9459.82	< 0.001
Total treatment cost ($)	4090.42 ± 1502.65	4364.48	12,620.88 ± 4242.65	11,693.87	< 0.001

$1.00 = ¥6.72 (March 11, 2019)

*$* US dollar, *¥* Chinese yuan, *ENPG* endoscopic nasopharyngectomy, *IMRT* intensity-modulated radiotherapy, *SD* standard deviation

## Discussion

We reported that the application of ENPG alone for localized stage I NPC and showed that ENPG achieved satisfactory oncological outcomes for localized stage I NPC, with a 5-year OS, LRFS, RRFS and DMFS all of 100%. Compared with the simultaneous period stage I IMRT-treated patients, ENPG presented with similar survival outcomes, but had better QOL, in terms of dry mouth and sticky saliva, and its total medical cost was cheaper, which indicated that ENPG might be an effective treatment for localized stage I NPC.

Radiotherapy has been recommended as the primary choice of radical treatment for NPC by the NCCN guidelines, and IMRT is the preferred method [17, 18]. However, oncologists are occasionally faced with patients’ refusal to radiotherapy and tendency to surgery. Some patients with satisfactory QOL might worry about radiation-related toxicities [19–22], which could have a relatively high acute severe mucositis rate of 32.5% [23] and late xerostomia rate of 61.5% [5], even for early stage NPC treated with radiotherapy alone. Additionally, severe toxicities, such as hyposalivation [20], dysphagia, skin and soft tissue damage caused by neck irradiation [19], could also compromise different aspects of the QOL of patients. In the current study, patients refused radiotherapies for different reasons, such as pregnancy, financial difficulty, claustrophobia. For these patients, surgical treatment was the preferred choice. However, only open surgery, such as the maxillary swing approach, transpalatal approach, and transmandibular transpterygoid approach, was used for recurrent NPC in the past [24]. Open surgery was often accompanied with high rates of complications, including soft palate dysfunction (54.8%), trismus (48.4%), secretory otitis media (64.5%), dysphagia (38.7%), nasal regurgitation (25.8%), and positive tumor margin (29.0%) [25], which was repellent for both doctors and patients. Minimal invasive surgery was needed but evidence of its performance in literature was lacking.

With the development of sinus endoscopy, EEA could be performed as a less invasive procedure for the removal of the nasopharyngeal tumor [26], however, there were still several limitations when applying the radical excision of NPC because of the inconvenience of operating through a narrow nasal cavity, difficulties in achieving en bloc excision, and problems in wound healing. To overcome these limitations, a technical system was established and successfully employed for recurrent NPC using endoscopic nasopharyngectomy combined with pedicle nasal septum and floor mucoperiosteal flap reconstruction [9, 10, 14]. Based on the technical system, ENPG broke through the limitations and achieved satisfactory survival outcomes and QOL in locally recurrent NPC [13]. This shows that ENPG could be technically feasible for primary localized stage I NPC.

In fact, many localized tumors can be cured by endoscopic surgery alone [27, 28]. Endoscopic surgery has been applied in therapy for early gastric cancer [29–31], colorectal neoplasia [32], glottic cancer [33] and achieved satisfactory outcomes. Among the success factors of surgery, patient selection is of great importance. The NCCN guidelines recommend esophageal, esophagogastric junction and gastric cancers within 2 cm in diameter and without invading deeper than the superficial submucosa, absence of lymphovascular invasion and clear tumor margins to apply endoscopic resection as a therapeutic method. In this study, a well-cooperated NPC research team was established to perform the selection and exclusion criteria and to address potential adverse events. The distance from the tumor margin to the internal carotid artery was defined as no less than 0.5 cm to decrease the risk of hemorrhage. Additionally, smaller size (≤ 1.5 cm in maximum diameter) was considered more suitable not only for the conveniences of the operation but also for less risk of regional or distant metastasis [34–37]. Considering the high rate of nodal metastasis in NPC, even with T1N0M0 patient which might had potentially nodal metastasis, we required the RPLN and CLN should be no more than 0.4 cm and 0.6 cm respectively for minimal axial diameter in MRI, and should be without central necrosis or groups of two or more lymph nodes. As a minimal axial diameter of 0.4 cm or larger was highly sensitive to the diagnosis of lateral retropharyngeal lymph nodes metastases, with a sensitivity and negative predictive value of 94.6% and 88.98% [38], and previous study revealed that 96.5% CLNs with a minimal axial diameter no more than 0.6 cm were regarded as negative according to PET/CT diagnosis [39]. If the RPLN and CLN were between 0.4 cm to 0.5 cm, 0.6 cm to 1.0 cm for minimal axial diameter respectively, or there was a controversary about the diagnosis of LN metastasis, PET/CT, core biopsy or excision biopsy and multidisciplinary consultation were to be conducted for confirmation. ENPG was performed after the CLN was proved to be negative.

As to the resection distance from the tumor margin, it was usually based on pathological evidence in other cancers, such as early invasive colorectal carcinoma [40], while it was rare in the past for NPC due to difficulties in acquiring general en bloc tumor specimens. In this study, we refer to the margin of CTV1 in the radiotherapy target, which was defined as GTV plus an additional anterior, superior, inferior, and lateral margin of 5 mm to 1 cm and an additional posterior margin of 2 mm to 3 mm. Further analysis of ENPG specimens might offer further guidance. The absence of local recurrence in these 10 patients indicates that the 0.5–1.0 cm margin may be enough for localized stage I NPC. Interestingly, we found that in the past, low-risk clinical tumor volume (CTV2) [15], which was defined as CTV1 plus a 0.5–1.0 cm margin, was commonly set in stage I NPC and required prophylactic irradiation of the bilateral upper neck. However, this CTV2 region and prophylactic irradiation of the bilateral upper neck were absent in all 10 patients who underwent ENPG alone in this study, but none of the patients experienced local relapse, regional relapse or distant metastasis. This phenomenon might bring some new insights to the principles of the radiotherapy target volume outline.

However, considering the novelty in applying radical ENPG for NPC, the high selection criteria of localized stage I cases and very limited number of patients included in this analysis, the clinical application of this procedure should be very prudent. Surveillance for recurrence or metastasis after treatment should be followed precisely, and cooperation in a multidiscipline team should be sought before clinical implementation. We have initiated a prospective clinical trial to further evaluate the safety and efficacy of ENPG (ClinicalTrials.gov number, NCT03353467) for localized early stage NPC.

## Conclusion

ENPG alone was associated with promising long-term survival outcomes, low medical costs, and excellent QOL for early stage NPC in our study, which may be an alternative strategy for newly diagnosed patients with localized stage I NPC who refuse IMRT.

## Data Availability

The key raw data have been deposited into the Research Data Deposit (http://www.researchdata.org.cn), with the Approval Number of RDDA2019001140 and the datasets used in this study are publicly available.

## Abbreviations

AJCC: American Joint Committee on Cancer
CTV: clinical tumor volume
DMFS: distant metastasis-free survival
EEA: endoscopic endonasal approach
ENPG: endoscopic nasopharyngectomy
EORTC QLQ-H&N35: European Organization for Research and Treatment of Cancer Quality of Life Questionnaire head and neck-specific
EORTC QLQ-C30: European Organization for Research and Treatment of Cancer Quality of Life Questionnaire-Core 30 general
GTV: gross tumor volume
ICRU: International Commission on Radiation Units and Measurements
IMRT: intensity-modulated radiotherapy
LRFS: local relapse-free survival
MRI: magnetic resonance imaging
NCCN: National Comprehensive Cancer Network
NPC: nasopharyngeal carcinoma
OS: overall survival
PET/CT: ^18^F-fluorodeoxyglucose positron emission tomography and computed tomography
QOL: quality of life
rNPC: recurrent NPC
RRFS: regional relapse-free survival
WHO: World Health Organization
